# S100 B: A new concept in neurocritical care

**Published:** 2017-04-04

**Authors:** Omidvar Rezaei, Hossein Pakdaman, Kurosh Gharehgozli, Leila Simani, Amir Vahedian-Azimi, Sina Asaadi, Zahra Sahraei, Mohammadreza Hajiesmaeili

**Affiliations:** 1 Skull Base Research Center, Loghman Hakim Medical Center, Shahid Beheshti University of Medical Sciences, Tehran, Iran; 2 Brain Mapping Research Center, Shahid Beheshti University of Medical Sciences, Tehran, Iran; 3 Clinical Research Development Center, Loghman Hakim Hospital, Shahid Beheshti University of Medical Sciences, Tehran, Iran; 4 Trauma Research Center, School of Nursing, Baqiyatallah University of Medical Sciences, Tehran, Iran; 5 Department of Clinical Pharmacy, School of Pharmacy, Shahid Beheshti University of Medical Sciences, Tehran, Iran; 6 Anesthesiology Research Center, Loghman Hakim Medical Center, Shahid Beheshti University of Medical Sciences, Tehran, Iran

**Keywords:** Biologic Marker, Surrogate Marker, Serum Marker, S100B Protein, Progressive Patient Car

## Abstract

After brain injuries, concentrations of some brain markers such as S100B protein in serum and cerebrospinal fluid (CSF) are correlated with the severity and outcome of brain damage. To perform an updated review of S100B roles in human neurocritical care domain, an electronic literature search was carried among articles published in English prior to March 2017. They were retrieved from PubMed, Scopus, EMBSCO, CINAHL, ISC and the Cochrane Library using keywords including “brain”, “neurobiochemical marker”, “neurocritical care”, and “S100B protein”. The integrative review included 48 studies until March 2017. S100B protein can be considered as a marker for blood brain barrier damage. The marker has an important role in the development and recovery of normal central nervous system (CNS) after injury. In addition to extra cerebral sources of S100B, the marker is principally built in the astroglial and Schwann cells. The neurobiochemical marker, S100B, has a pathognomonic role in the diagnosis of a broad spectrum of brain damage including traumatic brain injury (TBI), brain tumor, and stroke. Moreover, a potential predicting role for the neurobiochemical marker has been presumed in the efficiency of brain damage treatment and prognosis. However further animal and human studies are required before widespread routine clinical introduction of S100 protein.

## Introduction

In the recent past decades, various elements have been recommended as practical biochemical indicators of brain injury, including myelin basic protein, adenylate kinase, lactate, creatinine phosphokinase isoenzyme BB, S100B, neuron specific enolase, and glial fibrillary acidic protein (GFAP).^1^ Biochemical markers have been incrementally identified as potential accurate diagnostic tools. In theory, a biomarker should be accurate and accessible and have specific properties such as suitable predicting variables including high sensitivity, specificity, positive predicting value, positive likelihood ratio, area under the care, and low negative predictive value and negative likelihood ratio.^2^^,^^3^ Moreover, reproducibility and cost-effectiveness of analytical methods are other important properties.^3^ Recently, clinical use of bioneurocritical markers like S100 proteins has been evaluated and extensively increased. Researchers believed that astrocytes cultivate the calcium-binding peptide, which conducts an autocrine and paracrine effect on glial cells and neurons. The increased levels of S100B peptide can be detected in a variety of clinical or pathological injuries to the central nervous system (CNS).^4^^,^^5^ Due to the non-availability of various imaging techniques as gold-standard diagnostic tools and the need for immediate medical intervention to prevent permanent brain damage and disability, it seems plausible to seek alternative available methods. For this purpose, biomarkers can play a prominent role in a neurocritical care setting. The succinct review is an updated explanation of the role of S100B in human neurocritical care domain. 

**Search Strategy**

To perform an updated review on role of S100B in human neurocritical care domain, we carried an electronic literature search among articles published in English prior to March 2017. Articles were retrieved from PubMed, Scopus, EMBSCO, CINAHL, ISC and the Cochrane Library using keywords including “brain”, “neurobiochemical marker”, “neurocritical care”, and “s100b protein”. The integrative review included 48 studies until March 2017 that are presented and discussed as follow.

***Structure and functions of S100B protein:*** The Biologic marker, S100 protein, belongs to the family of Ca^2+^ binding proteins from chromosome 21.^   6 ^ The protein can help to regulate intracellular levels of calcium.^7^ Moore and colleagues were the first to name S100 as a protein in 1965, based on the characteristic of protein which is 100% solubility in a saturated solution with ammonium sulphate.^4^ Subsequently, two related homodimeric proteins S100A1 and S100B were identified.^7^ The former (consists of two α subunits) is mainly detected in kidney, neurons to muscle, and other organs. The second (consists of two β subunits), is rare and is found in neural glial cells and Schwann cells.

Nowadays, S100B protein has broad spectrum as minimally 25 biomarker proteins have been recognized that had similar structure to this protein.^4^ Together with other proteins of S100 family, S100B is located in the cytoplasm and nucleus of the astrocytes and conducts regulatory function of cytoskeletal structure and cell proliferation. Although it is significantly built in Schwann cells and astrocytes, the protein has been detected in other tissues such as bone marrow cells, chondrocytes, lymphocytes, adipocytes, and melanocytes. The protein is eliminated via renal excretion.^8^

S100B has been suggested to play a part in a variety of cellular processes, primarily via binding to key synaptic proteins and inhibiting their phosphorylation.^   9 ^ The extracellular form of S100B is physiologically involved in the development and maintenance of CNS homeostasis.^   3 ^ This form is synthesized and secreted by astrocytes.^10^^,^^11^ The mechanisms of regulating S100B secretion are not completely understood and appear to be related to different factors^12^^,^^13^ including interleukin β, the proinflammatory cytokines, metabolic stress,^   14 ^ and tumor necrosis factor alpha α1.^1   5 ^ Furthermore, a previous research suggested that S100B secretion involves the MAPK pathway and seemingly could involve NF-kB signaling.^13^ Within cells, it has several roles such as Ca_2_^+^ homeostasis, protein phosphorylation, and regulation of cell proliferation, transcription, differentiation, enzyme activity, and metabolism. When secreted into the extracellular medium, S100B exerts regulatory effects on neighboring cells i.e., astrocytes, neurons, and microglia.^   1 0^

S100B effects are closely dependent on its levels. At nanomolar concentrations and in vitro, it can enhance survival of neurons in various systems during development, and stimulate neurite outgrowth in cerebral cortex neurons.^16^ In vitro neurotrophic activity of S100B has been ascertained for neuronal cells in two points including the neuronal maturation and glial cell proliferation.^17^ Decreasing the loss of mitochondrial function and cell death are other effects of S100B.^16^ ^,^^18^

The roles of the protein in CNS development and recovery after injury are related to the neurotrophic and gliotrophic actions. Extracellular micromolar levels of S100B may have toxic effects.^19^ The protein at micromolar levels in vitro induces apoptosis and stimulates the expression of proinflammatory cytokines. The neurotoxic effects of S100B in vitro is mediated by induction of apoptosis in neurons.^19^ Astrocytes release nitric oxide that cause neuronal death in high levels of S100B.^16^ To the best of the knowledge, the mechanism of the effects may begin in two ways: by inducing raised levels of intracellular calcium and activating caspase-3 and by activation of inducible nitric oxide syntheses. ^20^^-^^24^ Besides the mentioned mechanisms, the receptor for advanced glycation end products (RAGE) pathways mechanism is also submitted.^22^^-^^26^ RAGE, a member of the immunoglobulin super family, can be bound by several ligands such as S100B. It is assumed that RAGE-mediated nuclear factor β activation can be responsible for the toxic (at high levels) and the pro-survival (at low levels) effect of S100B.      ^26^^,^^27^ S100B can also up regulate neuronal RAGE expression.^28^^,^^29^ S100B has short biological half-life (approximately 30-minute). However sustained high levels of S100B can be due to uninterrupted release from influenced and injured tissues.^30^^,^^31^ S100B has highest level in milk, cerebrospinal fluid (CSF), and serum. In addition, the protein can be detected and found in other fluids including amniotic fluid, urine, and in cord blood.^32^ Serum and CSF levels of S100B can be considered as a marker in postmortem and clinical investigations in brain damage field.^33^

**S100B: A suitable CNS diseases marker**

***Traumatic brain injury (TBI):*** Recently, S100B measurement in suffering patients from TBI has been interested among researchers because high levels of S100B in CSF and serum has been considered as marker of cell damage in human CNS after TBI.^34^ A review of the literature showed that there is a positive correlation between S100 parameters and intracranial pressure and cranial computed tomography (CT) findings.^   35 ^ For example, diagnosis of head trauma related lesions on CT scan with knowing the level of S100B with a cut-off of 0.1 µg/l is possible and the S100B level can be helpful in this clinical scenario (99% sensitivity and 30% specificity). Furthermore, knowing the level of S100B in clinical diagnostic protocol in minor head injury in accordance with cranial CT scan can decrease scanning by up to 30%.^36^

In severe TBI, S100B levels more than 1.13 ng/ml were correlated with high mortality (100% sensitivity and 41% specificity).^37^ In this regard, it was concluded that serum S100B demonstrates the severity of injury and improves the prediction of outcome after severe TBI.^9^      It is expressed that S100B level few hours post mild TBI is a suitable predictor for post-concussion syndrome in later times. A study showed that early S100B levels in 3 and 6 hours post mild TBI can possibly be informative in predicting some events about the outcome,^34^ because there was a positive correlation between the pathological findings of CT and high levels of S100B.^38^

In predicting the treatment efficacy post severe TBI, the evidence suggested that S100B protein is a sensitive biomarker.^39^ The biomarker plays a significant role in early predicting the development of intracranial pressure and mortality after acute brain injury. Furthermore, it is possible to identify secondary intracranial pressure increase with monitoring the S100B levels in risky patients and ultimately, to prevent from succeeding fatal outcome. The main goal of monitoring can optimize treatment protocol.^19^

Although according to the literature S100B level is not an FDA approved tool for clinical use, in mild TBI patients without significant extra-cranial injuries, S100B level less than 0.1 μg/l in four hours post injury may replace CT.^40^ In Scandinavia, serum S100B levels of 0.1 μg/l within six hours post injury in accordance with Glasgow Coma Scale score of 14-15 in patients without known risk factors were applied in the initial management of minimal, mild, and moderate head injuries. The patients can be discharged from the hospital without performing CT scan, although the level of evidence for management protocol is strong recommendation with moderate quality.^41^

**Brain tumors**

Elevated levels of S100B in neoplastic conditions such as astrocytomas have been spotted.^42^ It is assumed that S100B can monitor progression of cancer through inhibiting the function of tumor suppressor gene p53 with calcium dependent pathway.^   43 ^ For meningeal tumors, the involvement of S100B has been explored in two reports. The first report investigated serum S100B levels of 50 meningioma patients preoperatively and post-craniotomy for 7 serial days.^44^ Results showed that augmented S100B had high correlation with larger tumors, intraoperative difficulties, post-craniotomy acute degradation and long-term poor outcome. In addition, higher levels of S100B in postoperative period were associated with higher deterioration risk concurrent with poorer outcome. Moreover, the hypothesis that S100B levels can be used as an early biomarker in post-craniotomy brain damage in meningioma patients was verified.^45^

Stranjalis, et al. evaluated the connection between serial serum S100B protein measurements and post-craniotomy clinical worsening in patients with meningioma surgery.^   44 ^ S100B serum condensation in patients diagnosed with glioma have been surveyed in some studies.^46^ ^,^^47^ Gartner, et al.^48^ showed elevated plasma levels of S100B in the peripheral blood of two of three patients, at 11 and 13 months prior to the detection of a malignant glioma. Vos, et al.^49^ indicated notably shorter survival in patients with high serum S100B levels, and proposed that serum S100B protein may be predictive in variable cerebral gliomas. In addition, serum S100B levels are utilized for the early detection of recurrence of tumor or metastases.^30^

**Stroke**

Serum S100B levels have been extensively studied as a biomarker in acute ischemic stroke. In fact, the score of admission National Institute of Health Stroke Scale^50^ and total infarct volume^51^ had a positive correlation with S100B levels. Although the S100B levels in CSF are forty folds higher than serum, serum S100B level has been ascertained to significantly rise following ischemic stroke from 10 hours to 2-3 days from onset of stroke.^52^ In addition to the increasing S100B profile, serum S100B levels are helpful in distinguishing between nonvascular vertigo and posterior circulation strokes. The declaration was approved by two prospective observational studies that showed serum S100B levels in posterior circulation stroke were remarkably high.^53^ ^,^^54^ Detecting stroke in vertigo patients by serum S100B had excellent sensitivity (94.4%) and partially poor specificity (31.8%).^54^ One study reported that patients with transient ischemic attack or normal CT brain imaging at onset had notably lower serum S100B levels, with little alteration over time, compared to patients with an appreciable neurological deficit and abnormal brain imaging at the onset.^51^ Usually, the peak levels of serum S100B are during the first 3-4 day succeeding acute ischemic stroke^55^ but single serum S100B level in 48 and 72 hours following stroke attack can provide suitable prediction of infarct volume and functional outcome in non-lacunar middle cerebral artery infarction.^56^

The importance of considering serum S100B over time is reflected in the published results with different validities. A 12-hours serum S100B level more than 0.35 ug/l could predict the malignant infarction with good sensitivity and specificity (75% and 80%, respectively) while a 24-hours serum S100B level more than 1.03 ug/l could predict the event with higher sensitivity and specificity (94% and 83%, respectively).^57^ Several studies have shown that serum S100B levels measured in samples taken more than 24 h after stroke onset had a strong correlation with the degree of neurological deficit and the final infarct volume. Some clinical pearls can be concluded from raised levels of serum S100B after acute spontaneous intracranial hemorrhage that are "worse early", "later evolution”, and "strict correlation with initial hematoma volume".^58^ After ischemic and hemorrhagic strokes, S100B protein is released into the blood. There is good association between the release pattern of S100B and volume of vascular lesion. S100B protein is a sensitive biomarker of brain injury following stroke. It is probable to employ serum S100B level in monitoring treatment for stroke.^59^ In stroke patients, there was a regular increase in S100B 1-3 days after the onset of symptoms followed by slowly decreasing values as already depicted by others.^60^

In subarachnoid hemorrhage (SAH), the serum S100B levels are helpful and can help clinicians to examine the hemorrhage severity because there is a strong positive correlation between serum S100B values and initial SAH severity.^61^^,^^62^ The initial SAH severity has been studied by Fisher score in para clinical settings^63^ and Federation of Neurological Surgeons grading scale in clinical settings.^64^ It can be expressed that S100B levels in CSF and blood are excellent predictors of SAH outcomes in affected patients.^65^ ^,^^66^ For example, daily mean value of S100B more than 0.4 μg/l have a remarkably poor prognosis.^   67 ^ S100B and GFAP following spontaneous SAH have strong positive correlation with neuroimaging and clinical severity of the disease. The clinicians can use the correlation in better initial and late assessments,^   45 ^ and even to determine the outcome.^65^ Higher levels of S100B in SAH patients is concurrent with indeterminate prognosis and inadequate outcome.^11^

S100B has a broad spectrum of clinical and paraclinical use in SAH, cerebral infarction, and intracranial hypertension. However, vasospasm as SAH secondary complications can be diagnosed or predicted by neither serum nor CSF S100B level.^68^

## Conclusion

New biomarkers of CSF and serum have been evaluated to improve diagnosis and predict outcome more accurately. Among them the S100B protein is a suitable CNS neurobiochemical candidate for diagnostic, prognostic, and even therapeutic purposes. However, there are risk of false positive and false negative laboratory errors and should be used with caution. Further animal and human studies are required before widespread routine clinical introduction of S100 protein.

## Notes:

**How to cite this article: ** Rezaei O, Pakdaman H, Gharehgozli K, Simani L, Vahedian-Azimi A, Asaadi S, et al. S100 B: A new concept in neurocritical care. Iran J Neurol 2017; 16(2): 83-9.
